# The tip of the iceberg: Profiling cooling agents using computational approaches to inform tobacco regulatory science

**DOI:** 10.1371/journal.pone.0346126

**Published:** 2026-04-16

**Authors:** Saibal Chakraborty, P. Dilip Venugopal, Maria Kaltcheva, Jueichuan Connie Kang, Reema Goel

**Affiliations:** 1 Division of Product Science, Office of Science, Center for Tobacco Products, US Food and Drug Administration, Silver Spring, Maryland, United States of America; 2 Division of Nonclinical Science, Office of Science, Center for Tobacco Products, US Food and Drug Administration, Silver Spring, Maryland, United States of America; Universidad Autonoma de Chihuahua, MEXICO

## Abstract

Cooling agents (chemicals added to impart a cooling sensation) in tobacco products are receiving increased attention due to their use as menthol substitutes. However, detailed information on their chemical structures, physico-chemical properties, cooling activity, and human and ecotoxicity potential has not been compiled. Our goal was to profile cooling agents that could be used as menthol substitutes in tobacco products, along with descriptions of their cooling properties and estimated toxicological effects. First, we compiled a library of 228 cooling agents, including 180 unique two-dimensional (2-D) chemical structures based on a review of multiple public data sources (literature, invention patents, and public databases). The library includes chemicals with a “cool” and/or “mint” flavor profile as designated by the Flavor and Extract Manufacturers Association (FEMA), chemicals reported to activate the cation channel transient receptor potential melastatin 8 (TRPM8) receptor, and/or chemicals with reported subjective perceived sensory cooling. Second, we classified the cooling agents into three main chemical skeletons using similar structural motifs and estimated their physico-chemical properties related to cooling attributes using Schrödinger Canvas software, including log octanol-water partition coefficient (LogKow), vapor pressure, and electrotopological state index. Third, to investigate the cooling agents’ similarity to each other and to menthol, we applied an unsupervised machine learning algorithm (Knowledge Discovery by Accuracy Maximization [KODAMA]) and hierarchical clustering techniques and identified six clusters. Fourth, we compiled available perceived cooling intensity data. Fifth, we used models based on experimental and predicted data (EPA’s web-based Hazard Comparison Module [HCM]) to estimate human health toxicity and ecotoxicity. Some cooling agents are associated with genotoxicity, developmental toxicity, skin irritation, and eye irritation, and pose toxicity to fish, algae, and invertebrates. Using this surveillance approach will help inform future tobacco regulatory decisions and policies by profiling chemicals used as cooling agents in tobacco products and those that pose potential health hazard concerns.

## Introduction

The Family Smoking Prevention and Tobacco Control Act authorizes the US Food and Drug Administration’s (FDA’s) Center for Tobacco Products (CTP) to regulate tobacco products, which involves reviewing new tobacco product applications to ensure that marketing the new products is appropriate for the protection of the public health [1–3]. Furthermore, as part of new tobacco product review, FDA evaluates the environmental impact of its tobacco regulatory actions per rules described in 21 CFR § 25 as mandated by the National Environmental Policy Act [4]. A major component of this evaluation is the comprehensive analysis of tobacco product ingredients’ impacts on public health and the environment, including effects related to product appeal, toxicity, and addictiveness [5]. One category of tobacco product ingredients that has recently received increased attention is cooling agents. Cooling agents are chemicals with physiological cooling properties that impart a cooling sensation to the skin and mucous membranes of the mouth, nose and throat, although no actual physical cooling takes place [6]. Such cooling agents are of great interest to manufacturers for use in cosmetics, food, and tobacco due to their anesthetic, soothing, and refreshing effects [7]. Chemically induced trigeminal somatosensory cooling (termed chemesthesis), which occurs when these chemicals are ingested or inhaled, represents a distinct sensory pathway separate from traditional taste and smell mechanisms [6].

Menthol is the most well-known and widely used cooling agent. Menthol can be found in many types of tobacco products, including cigarettes, e-cigarettes/electronic nicotine delivery systems (ENDS), waterpipe, and smokeless tobacco [8–11]. Along with sensory cooling properties, menthol produces a characteristic minty odor (olfactory) and often perceived as a ‘minty taste,’ though this sensation is primarily trigeminal rather than gustatory [6]. Mentholated tobacco products have been reported to be gateway products, which facilitate experimentation by nonsmokers and progression to regular smoking and dependence and which are harder to quit [12]. Due to menthol’s notable effects and wide product applicability, many efforts to identify similar cooling agents have been undertaken. For example, in the 1970s, Wilkinson Sword Ltd. (WS) conducted extensive research on menthol derivatives based on modifications to the menthol structure and evaluated about 1200 WS chemicals for their cooling properties [13,14].

FDA has proposed two tobacco product standards that would prohibit menthol as a characterizing flavor in cigarettes [15] and cigars [16]. When finalized, these two tobacco product standards could significantly reduce the disease and death from combusted tobacco product use, as indicated by recent simulation studies [17,18]. Countries including Denmark, Netherlands, and Germany and an increasing number of US states such as California, Maine and New York have imposed restrictions on mentholated tobacco products [19]. It has been reported that the tobacco industry has responded to such sales restrictions by introducing and marketing “non-menthol” tobacco products [20,21]. For instance, the presence of cooling agents such as WS-23 and WS-3 in tobacco products has been recently reported [9–11,20,22] The new Surgeon General report provides an overview of the multisensory experiences associated some cooling agents that can contribute to tobacco-related health disparities [23].

Similar to menthol, such cooling agents are expected to contribute to tobacco product appeal, tolerability, and dependence [10,24–26]. Factors other than minty taste enhance mentholated tobacco products’ appeal to youth and young adults. For example, menthol can reduce irritation of oral and pharyngeal membranes and diminish pain elicited by tobacco chemical stimuli [27,28]. Similar to menthol, many cooling agents can also reduce airway irritation, coughing, and obstruction [29]. Internal tobacco industry documents made public through litigation against tobacco companies, as researched for this work and also documented by others [10,11,20,22], indicate that the industry continues to add novel cooling agents to maintain a diverse tobacco product portfolio, retain menthol users, attract new consumers, and/or evade regulations. The number of chemicals available for use as cooling agents continues to grow with innovations in flavor chemistry [14,30]. As reflected by ever-increasing patent approvals, ongoing efforts to discover new cooling agents are driven by a desire to have a repertoire of chemicals with differing characteristics that can be used in combination to provide users with a favorable cooling sensation in a variety of consumer products.

As part of consumer industry efforts to develop and identify new cooling agents, different approaches are used for assessing agents’ cooling attributes. One of the most well-established and understood mechanisms of a chemical’s physiological cooling property relates to its activation of calcium-permeable cation channel transient receptor potential melastatin 8 (TRPM8) [31]. The cooling potency of a chemical can be quantitatively measured by half maximal effective concentration (EC50) of TRPM8 activation. However, TRPM8 receptor-binding studies are unable to provide information on the quality of human sensations (e.g., odor, taste) these chemicals evoke. In addition, TRPM8 receptor binding potency provides some information on maximum cooling intensity but has little correlation with the duration of the effect [7]. Descriptive sensory perception studies by trained panelists are another approach to assess cooling attributes of chemicals [6]. Cooling agents in these studies are often compared to menthol with regard to their odor and taste profiles, cooling strength, time of cooling onset, and duration of cooling effect. Thus, although some cooling agents can be grouped based on similarity/dissimilarity to the menthol chemical structure, the structural relationship to cooling activity (TRPM8 activity or sensory perception) is not clearly understood [7]. The in-vitro, cell-based TRPM8 receptor binding studies and human sensory perception studies described above are expected to correlate well [32]; however, no studies have examined this correlation directly.

The plethora of existing cooling agents, the continued emergence of new cooling agents, and the wide use of menthol and other cooling agents in tobacco products make understanding the associated risks of this ingredient category a public health priority. A recent review [10] focusing on a few WS chemicals reported that very limited experimental toxicological data are available on cooling agents, but available data do indicate some associated toxicity in the lungs, kidneys, and liver [33–36]. In addition, the ecotoxicity potential of cooling agents is currently unknown. Although the WS chemicals initially tested were menthol derivatives, gradually, chemicals showing cooling properties and with structures completely unrelated to menthol were synthesized and patented. There are limited data available to describe these cooling agents’ chemical structural landscape, cooling properties, and toxicity. Knowledge of such information will enhance our understanding of the potential toxicological hazards of cooling agents in tobacco products. In the absence of experimental data, in this study we use computational (in silico) and new approach methodologies that can help identify suspect toxicants, toxicity pathways, and potential health effects of a large number of chemicals in a time and resource efficient manner [37]. We also used computational models to evaluate chemical structures and to analyze the physio-chemical properties of the cooling agents to provide a comparison to menthol.

In this study we (1) compile a library of cooling agents with any reported cooling attributes (FEMA flavor profile, TRPM8 activity or sensory cooling perception) (2) estimate the physico-chemical properties of the cooling agents in the library using computational (*in silico*) evaluation, (3) understand similarities and dissimilarities of cooling agents to menthol by clustering the chemicals based on structures, (4) compile available perceived cooling intensity data, and (5) compile hazard data on cooling agents for human and environmental toxicity endpoints based on available empirical and computational predictive data.

## Results

### Cooling agent library components

The cooling agent library consists of 228 chemicals, including stereoisomers for some chemicals (**Supplemental Data File)**. The type of information and number of chemicals associated with each data source that was collated in the cooling agent library is depicted in **Table 1**. Wherever available, stereoisomer-specific chemical data is included from different sources in the cooling agent library to capture any stereoisomeric-specific data for cooling or toxicity. For example, menthol has eight possible stereoisomers with different cooling intensity [38]. The cooling agent library with overall 228 chemicals contained 180 unique chemicals based on 2-dimensional molecular structure. In the library, 121 cooling agents have a designated FEMA number, with a subset of 101 chemicals specifically encompassing cool/cooling/mint/camphor FEMA profiles. Cooling activity, as reflected by TRPM8 binding and sensory perception could be retrieved for 34 and 56 chemicals, respectively. A search for potential tobacco additives identified 61 cooling agents that were considered for use in any tobacco product – such as cigarette, pipe tobacco, cigar, smokeless tobacco, and/or ENDS e-liquids. Our data indicate that cooling agents have a wide range of physico-chemical properties compared to menthol (**Table 2**).

**Table 1:** Information sources and associated number of chemicals available for cooling agents in the library. Note that as different types of cooling agent-related information are presented here, the numbers do not add up to 228 overall chemicals.

Cooling Agent Library	Number of chemicals
Cooling Agents	228
Subset with Unique 2D *structures*	180
Flavor and Extract Manufacturers Association of the United States (FEMA) number	121
Subset with cool/cooling/mint/camphor FEMA profiles	101
Transient Receptor Potential Melastatin 8 (TRPM8) receptor binding data	34
Considered for Use in Tobacco Products	61
Sensory subjective cooling perception data	56

**Table 2:** Summary of salient computationally predicted physico-chemical properties of menthol in comparison to cooling agents in the library based on 180 unique 2-D chemical structures.

Physico-chemical Property	Menthol	Range in Cooling Agent Library
Molecular weight (Da)	156	84.1–409
Water solubility (mg/L)	434	6.49E-5 – 1E6
Vapor pressure (Antoine method, mm Hg)	0.0092	2.27E-15 – 1.29E1
Log octanol-water partition coefficient (log Kow)	3.38	−1.45–8.92
Electrotopological State Index	21.83	14.67–63.00

### Cooling agent chemical space and chemical clustering

**Fig 1:**
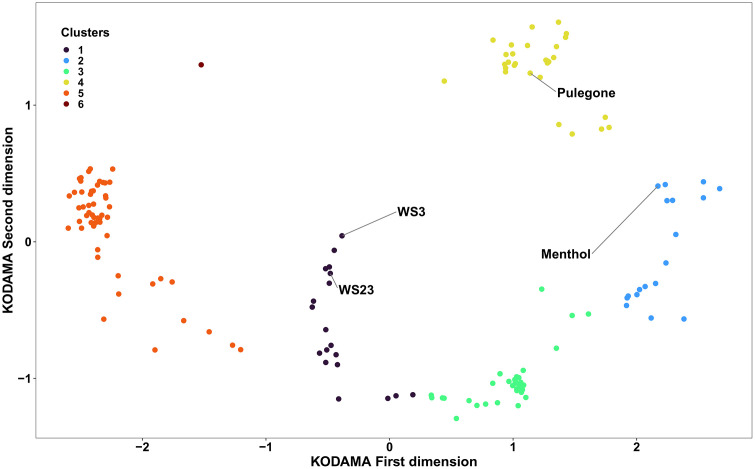
Cooling agent clustering based on chemical structures with some of the common cooling agents labelled. Structural information (SMILES) for 180 unique 2-D structures for cooling agents was clustered using KODAMA unsupervised machine learning and hierarchical clustering techniques to reveal structural similarity/dissimilarity. Additional details on the number of chemicals, representative structure, and type of cooling agents in each cluster are provided in **Fig 2**.

**Fig 2:**
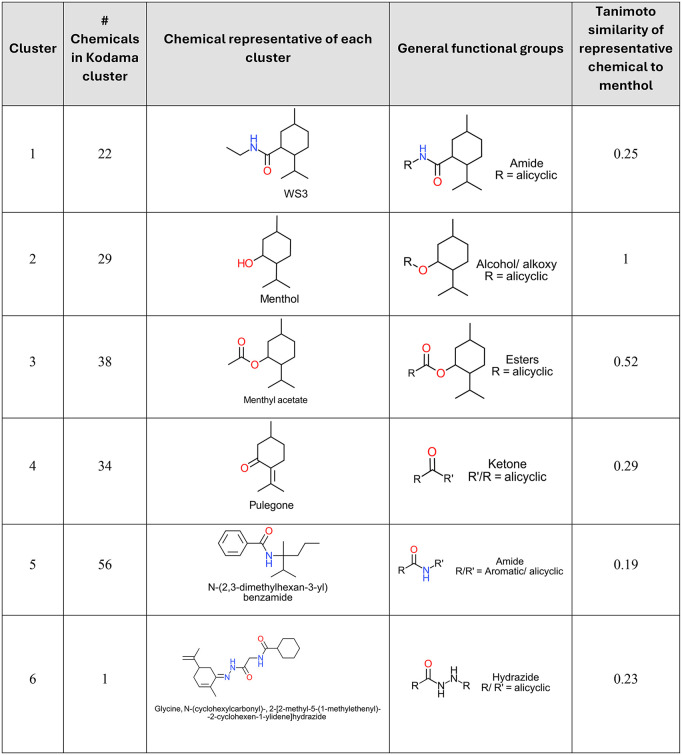
Structural and Chemical Information Associated with the Cooling Agent Clusters for the 180 unique 2-D structures based on the KODAMA clustering approach depicted in Fig 1.

KODAMA clustering enabled the visualization of the structural similarity/dissimilarity among the cooling agents. We found that the 180 unique 2-D structures segregated into six clusters visualized by plotting the KODAMA axes scores **(**Fig 1), which helps uncover patterns based on structural similarity/dissimilarity. Menthol was classified in cluster 2 along with other structurally similar chemicals. **Fig 2** provides information on the number of chemicals, representative structure, and type of cooling agents in each cluster. Broadly, each of the clusters based on KODAMA analysis constitutes indicative functional chemical groups (e.g., ketones for cluster 4, aromatic amides for cluster 5; see Fig 2). The cooling agents were primarily found in five clusters, while Cluster 6 included a single chemical with the hydrazide functional group.

### Cooling characteristics of cooling agents

TRPM8 receptor binding data and perceived sensory cooling intensity data were available for 34 and 56 cooling agents, respectively, with only 17 cooling agents having overlapping data. L- Menthol one of the best-studied TRPM8 activators, has a reported EC50 range of 2.2–185 μmol/L. However, due to the different methodologies in the in vitro TRPM8 binding assay, including differences in cell types overexpressing TRPM8 as well as detection probes, combining EC50 data from different studies is not ideal. The reported EC50 ranges of WS-3 and WS-23 are 3.7–5.9 and 2.2–44 μmol/L, respectively, reflecting a binding potency to TRPM8 that is similar to or lower than L-menthol [32,39].

**Fig 3:**
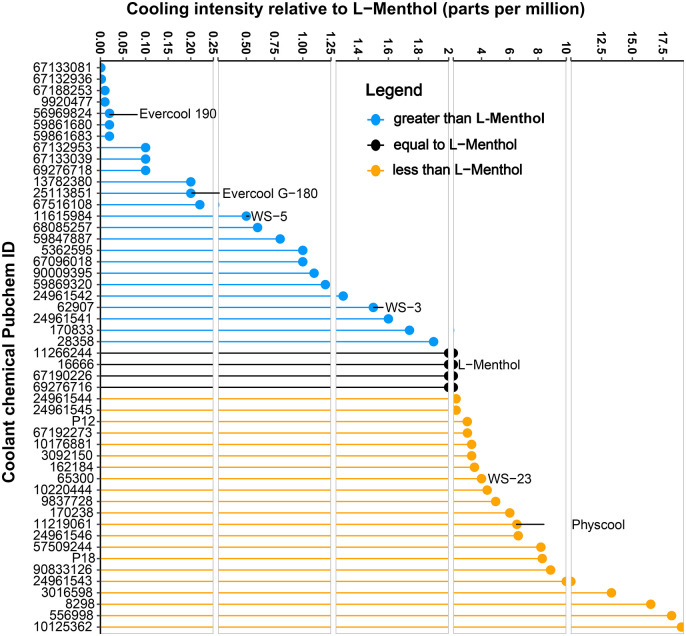
Comparison of perceived sensory cooling intensity of select cooling agents relative to L-menthol. The figure depicts the broad range of chemical concentrations (low values represent high cooling intensity) and those that give the same cooling sensation as L-Menthol (2 parts per million). The unique 2-D structures (49 chemicals) with reported cooling intensity are plotted with identified PubChem IDs, and a few also labeled by their common names (also see **Supplemental Data File)**. The grey vertical lines denote shifts in axis scale.

The sensory threshold concept can be used to characterize new cooling agents; the minimum intensity of stimuli perceived by the panelist is recorded and then a geometric mean of all panelists is used as the threshold value. In sensory perception studies, L-menthol has a reported cooling effect lasting 35 minutes, with a cooling threshold of 0.8–1.8 parts per million (ppm) by taste dilution, an odor threshold of 0.1–0.4 ppm, and a predominantly minty taste and odor [38]. The perceived sensory cooling intensity of a chemical (measured as “isointensity to L-menthol”) is defined as the concentration of a chemical that gives the same cooling sensation as 2 ppm of L-menthol [7]. Because the cooling intensity of a chemical is available relative to cooling from L-menthol (2 ppm or as100 strength), we were able to compile data **(Supplemental S1 Data File)** across studies/patents; i.e., concentration (in parts per million) representing cooling intensity of a given chemical. **Fig 3** depicts the broad range of reported perceived cooling intensity for 49 unique 2-D structure cooling agents in comparison to L-menthol, with up to 2000 times perceived cooling intensity than L-menthol. Shown in the figure for example, 1.5 ppm of WS-3 is similar cooling intensity as 2 ppm of L-menthol reflecting 1.3 fold higher perceived cooling than L-menthol. Besides cooling intensity, we captured available qualitative information on cooling agents’ organoleptic profile by the reported perceived odor and taste. Minty odor and minty taste were noted for 36 and 29 chemicals, respectively. Our data analysis shows that panelists were unable to detect any strong minty smell, or any taste usually associated with menthol for 71 and 47 chemicals, respectively. Sensory odor and taste data were not identified for 116 and 151 chemicals, respectively. We were also unable to find any trends in correlation between TRPM8 receptor binding affinity and sensory cooling intensity due to a lack of reported data.

### Toxicity and hazard assessment of cooling agents

**Fig 4:**
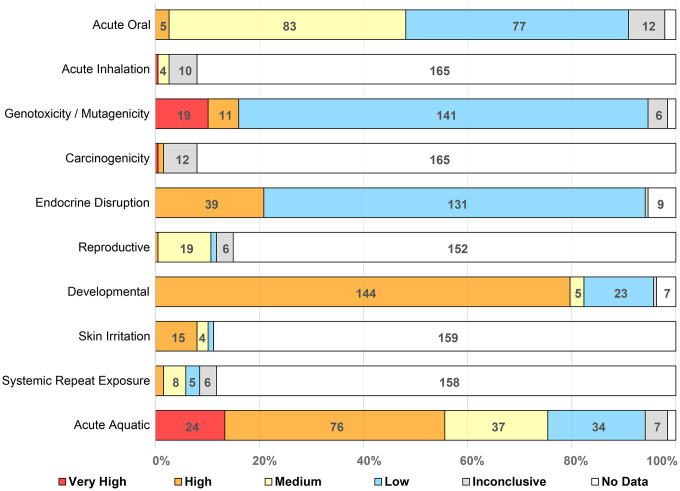
Summary hazard scores for the 180 cooling agents with unique 2-D structures for 10 toxicity endpoints relevant to tobacco exposure routes. These hazard scores were generated using EPA’s web-based Hazard Comparison Module, which compiles experimental and quantitative structure-activity relationship (QSAR) predictions data from multiple data sources.

We compiled hazard scores for chemicals using EPA’s web-based Hazard Comparison Module (HCM) to allow for a scoping assessment of the relative hazards across the human health effects and ecotoxicity. We found that the hazard records available, and thus the associated hazard scores, varied across different endpoints and chemicals. For five endpoints (acute mammalian oral toxicity, genotoxicity and mutagenicity, developmental toxicity, endocrine disruption, and acute aquatic toxicity), hazard profiles were available for more than 94% of the cooling agents. Information for the other human health and chronic aquatic toxicity endpoints was limited or “data-poor,” with hazard reports available for 15.5% or less of the chemicals. **Fig 4** summarizes the hazard scores for 9 human toxicity and 1 ecotoxicity endpoints. The toxicity endpoints specifically emphasize tobacco-relevant exposure routes, as route of exposure is a key determinant of toxicological outcomes. Overall, the results highlight that hazard scores varied across endpoints and chemicals. **Fig 4** also shows chemicals with no hazard records for endpoints to highlight the variation in data availability. For example, acute inhalation as well as carcinogenicity profiles were not available for 165 cooling agents. The complete hazard profiles for all the 15 human toxicity and the 2 ecotoxicity endpoints and their associated hazard records for the 180 unique 2-D structure chemicals generated using HCM are provided in the **Supplemental Data File**.

Next, in order to explore toxicity endpoints that may be most affected by cooling agents, for each endpoint we calculated the proportion of the chemicals with high and/or very high hazard scores. Of the five data-rich endpoints, the percentages of chemicals with high and/or very high hazard scores were: 83.2% for developmental toxicity (144/173), 56.2% for acute aquatic toxicity (100/178), 22.8% for endocrine disruption (39/171), 16.9% for genotoxicity and mutagenicity (30/177), and 2.8% for oral acute mammalian toxicity (5/177). We also identified the chemicals that have multiple high and/or very high hazard scores across all endpoints and found that 68 cooling agents have two or more high and/or very high hazard scores across all 15 human health effects.

## Discussion

In this paper, we compiled and analyzed publicly available information on 228 cooling agents and profiled their attributes (structural data, physico-chemical properties, sensory perceptions, TRPM8 binding, and toxicity hazard profiles). We first compiled and organized the cooling agents by identity and structure. The 228-cooling agent library includes 180 unique 2-D structures, with chemical stereoisomers for the most well-recognized cooling agents, such as menthol. For example, menthol has three chiral centers leading to eight possible stereoisomers (4 enantiomeric pairs) [38]. Compared to the other stereoisomers, L-menthol has the most intense cooling properties. L-Menthol is the predominant stereoisomer reported in tobacco products and no evidence of thermal racemization of menthol upon smoking has been found [40]. L-Menthol binds to TRPM8 receptor cavity using its hydroxyl and isopropyl groups [41]. Even the distinct spatial orientation of hydroxyl and isopropyl groups in menthol stereoisomers differentially affects binding and activation of the TRPM8 receptor, resulting in differential cooling sensation by the stereoisomers [28,40]. However, our structural analysis plan required the 2-dimensional molecular SMILES structural representation to calculate the physico-chemical properties, which do not take stereospecificity into consideration. In addition, stereoisomeric-specific data related to cooling intensity or toxicity for the majority of chemicals were lacking. Hence, for this work, data for physico-chemical properties and toxicity potential were estimated for the 180 unique 2-D chemical structures without considering any stereoisomer differences.

Our library contains 137 cooling agents with a p-menthane skeleton (including substituted p-menthane and menthol) based on their chemical structures. After compiling the cooling agents and structures, we further used a computational approach to determine structural similarity. This approach resulted in grouping cooling agents into six clusters. Clustering by KODAMA depicts structural diversity across the library **(Fig 1)**. Other clustering approaches based on grouping physico-chemical properties by cluster analysis or principal component analysis (PCA) may also depict the dataset. However, KODAMA, a machine-learning algorithm for feature extraction from noisy and high-dimensional data, offers improved dimensionality reduction compared to PCA or other multivariate methods [42,43]. Accordingly, KODAMA analysis enabled identification of groups of cooling agents represented by different chemical classes that are structurally different from menthol [44]. Review by a chemist who compared the cooling agent grouping by functional groups and by the KODAMA clusters found general consensus in terms of indicative functional chemical groups among most of the chemicals in each cluster. It is important to note that KODAMA clustering is dependent on the specific chemical structures and the total number of chemicals included in the dataset. Therefore, the identified clusters reflect structural relationships within this particular library and should not be interpreted as fixed or universally generalizable chemical groupings.

We conducted an exploratory analyses to assess whether cluster grouping aligned with available cooling or toxicity data. However, due to incomplete and heterogeneous endpoint data across the 180 unique 2-D structures, no consistent cluster-level generalizations could be drawn. Cluster 2 contains menthol and related chemicals sharing similar core structural features. Cluster 6, which contains a single hydrazide compound. Its separation solely reflects structural dissimilarity relative to other chemicals in the library. Given the dataset-dependent nature of unsupervised clustering and the limited data available on cooling activity, receptor interaction, or toxicity for many chemicals, cluster grouping should be interpreted as descriptive rather than predictive.

The preferred characteristics and properties of cooling agents intended for use in tobacco products, especially inhaled and combusted products, may differ from those in other consumer products such as such food and beverages, cosmetics and personal care items. For example, menthol, a solid with high vapor pressure at room temperature, is very soluble in alcohol and slightly soluble in water. Menthol is added to cigarettes in a variety of ways (e.g., sprayed as a solution on the cut tobacco during blending; placed in a capsule in the filter), and it can diffuse throughout the cigarette due to its high volatility [45]. However, menthol’s high volatility (as indicated by vapor pressure) also contributes to chemical loss by diffusion during cigarette storage. Non-menthol cooling agents with low volatility (such as WS-3, WS-5, WS-14 and menthane carboxamides) may reduce migration and potentially improve product shelf-life [45]. Carbonate esters of menthol, such as Frescolat® type MGC possess intrinsic cooling activity and remain stable in tobacco during processing and storage but can also thermally decompose during combustion to release menthol [45]. In our review of internal tobacco industry documents, we identified chemicals such as monomenthyl maleate and menthyl-4-hydroxyvalerate that have been described as menthol precursors, capable of releasing menthol upon thermal decomposition during combustion [46]. Chemicals whose sole documented function is thermal decomposition to menthol (without intrinsic cooling activity independent of menthol release) were excluded from the library. The structural variability across cooling agents in the library could lead to differences in physico-chemical characteristics. One of the objectives of this manuscript was to provide with estimates of some physico-chemical properties of cooling agents in comparison to menthol. Our analysis for 180 unique 2-D structures shows that the predicted physico-chemical properties varied widely across cooling agents. Experimental physico-schemical data is available for only a few chemicals. Besides, the experimental physico-chemical data will vary based on the different testing parameters. The Canvas software was used to predict physico-chemical properties based on unique 2-D structures. Providing details and discussions on the modeling parameters and optimization approaches for each physico-chemical parameter is outside the scope of this manuscript. Future research could more closely investigate how cooling agents’ physico-chemical properties under different experimental conditions and using different modeling approaches affect their selection for incorporation into different tobacco substrates and how they affect transfer rates across different tobacco products.

Of the 228 cooling agents included in our library, 61 have been documented in publicly available ingredient disclosures, patents, or internal industry documents as having been used or considered for use in tobacco products [47,48]. Our analysis revealed no clear pattern in structural features or physico-chemical properties to rationalize a criterion for selecting these particular 61 cooling agents in tobacco product development. Theoretically, hundreds of diverse cooling agents are available in the pipeline for use as tobacco product ingredients. Multiple cooling agents can be added at levels subthreshold (non-detectable in taste sensation) to consumers [49]; thus, there has been a call to ban menthol and all cooling agents as tobacco additives, rather than menthol as a characterizing flavor [19]. Furthermore, although odorless and tasteless cooling agents may be present in tobacco products [50], currently, other than the characterizing flavor of the tobacco product, tobacco manufacturers do not provide the intended flavor description nor the sensory profile for individual flavor ingredients added to their tobacco products for marketing in the US.

Descriptive sensory perception studies can provide information on a chemical’s temporal cooling sensory profile, including onset time, relative intensity, and duration of cooling effect. The cooling agents in the library differ in their sensory properties. Of note, 43 chemicals have no prominent taste or odor. Furthermore, ingredient concentration can change the sensory property. For example, at high concentrations, menthol can have an unappealing strong minty odor and minty taste, or create stinging, burning, bitterness, and sensory irritation [13]. The tobacco industry’s projects “cool without menthol” [51] and “sword” [52] were meant to target all smokers, including non-menthol smokers, with non-menthol cooling agents by separating the chemical’s sensory cooling from other sensory effects to attract a broader consumer base. These projects proposed that non-menthol cooling agents would still mask the harshness and irritation of the tobacco product, thereby reducing unpleasant experiences that may deter new users from repeated experimentation. Our results document that many chemicals have higher perceived sensory cooling compared to menthol (**Fig 3**), some up to 2000-fold. The perceived sensory cooling intensity may be targeted for increase with continued innovation and discovery. It is interesting that WS cooling agents have been reported in high nicotine concentration e-liquids to reduce throat irritation [53] and in “light” combustible cigarettes to maintain product appeal [53].

Both TRPM8 testing and sensory panel studies are methods used to evaluate a chemical’s cooling activity. TRPM8 receptors are widely expressed in sensory nerves and epithelial cells across the mucosa, skin, and viscera of the oronasal cavity. TRPM8 activation by chemicals established that these receptors can physiologically, and at a molecular level, detect chemicals with cooling properties. Using this testing approach, TRPM8 receptors have been found to be potently activated by different classes of chemicals such as p-menthane carboxamides, aliphatic/cyclic alcohols/esters/amides, heterocyclics, and keto-enamines [31]. However, despite the importance of chemical binding data to the TRPM8 receptor as an indicator of the chemical’s physiological cooling property, only a few studies were available, covering only 34 of the 228 cooling agents in our library. Recently, non-menthol structures such as WS-12 and icilin have been shown to induce different conformational changes in TRPM8 compared to menthol [41]; however, for most cooling agents with a variety of different structures, it is not yet clear how and where they bind to the TRPM8 receptor. Combining chemicals with distinct TRPM8 binding and activation sites could result in prolonged and intense cooling to enhance the consumer sensory experience [7]. The relative amounts of the primary and secondary “effect-enhancing” cooling agents may vary over a wide range of tobacco products depending upon the cooling effect desired [47]. For example, succinate-based cooling agents provide a cooling effect in a different area of the mouth and throat than menthol or carboxamide-based cooling agents [47]. Menthol can also interact with nicotinic receptors to modulate their activity allosterically and can increase nicotinic receptor expression in the brain [12,54,55]. In combination with menthol’s flavor and sensory effects, its effects on nicotinic receptors may play a role in facilitating experimentation and progression to regular smoking and hindering the ability to quit [12]. Due to the structural similarity of many cooling agents to menthol, it is plausible that some interact with the nicotinic receptors. However, there are very limited data evaluating nicotine receptor interaction with cooling agents.

Another difficulty in analyzing cooling agent data lies in the challenge of correlating in vitro cell-based TRPM8 receptor binding assay results with human perceived sensory cooling intensity study data. Of the 228 chemicals included in our library, TRPM8 binding data were identified for only 34 chemicals, and sensory cooling intensity data were available for 57 chemicals. Moreover, these study data were generated using different assay systems, methodologies, and laboratory conditions, limiting the ability to perform rigorous quantitative comparisons across chemicals. For example, some data indicate that WS-12 may be more potent at TRPM8 activation than WS-5 [29,31,56], yet WS-12 may exhibit lower perceived cooling intensity and slower onset of action compared to WS-5 [29,32]. These observations indicate that in vitro TRPM8 binding affinity/potency alone is insufficient to predict human cooling perception. A testing approach that integrates sensory panel studies and standardized receptor-based assays will provide the most comprehensive assessment of individual cooling agents.

Cooling agent toxicity is a significant factor contributing to the potential toxicological risks associated with tobacco products. Our library contains a subset of 121 chemicals with a FEMA number, including 101 chemicals encompassing cool/cooling/mint/camphor FEMA profiles. Historically, some tobacco manufacturers have marketed and sold tobacco products containing flavor chemicals considered by FEMA as “Generally Recognized As Safe” (GRAS) [57]. The GRAS determination only applies to chemicals evaluated under scientific procedures or commonly used in food under the conditions of their intended use. As discussed in detail in a recent review, the use of “GRAS” on the label of tobacco products to infer safety is scientifically flawed [58].

Our analysis indicates that toxicity data – and certainly high-quality data – are lacking for several human and environmental toxicity endpoints for many of the cooling agents in our library. Furthermore, the inhalation toxicity potential of most of the cooling agents is currently unknown. We utilized computational models, a widely accepted approach, to investigate areas that are data-poor. Several public and commercial models for predicting different endpoints have been built using different chemical datasets, molecular descriptors, and statistical methods. For this analysis, we used HCM, which compiles and integrates chemical hazard data across several human health and ecotoxicity endpoints for a batch of chemicals. HCM is useful as a screening tool for hazard identification due to the broad and rapid assessment of chemical toxicity potential. HCM provided a rapid screening approach across multiple human and environmental toxicity endpoints to help identify chemicals for further hazard assessment (**Fig 4**). One of the goals of this manuscript is to provide a scoping assessment of toxicity concerns from cooling agents rather than a detailed assessment for any particular endpoint or cooling agent. Screening-level hazard assessments identified several specific cooling agents of potential toxicological concern. Our work also highlights the need for work on “data-poor” endpoints (e.g., carcinogenicity and inhalation toxicity) as well as many “data-poor” cooling agents that could be used as menthol substitutes, particularly in the context of inhaled tobacco product use. Additionally, although stereospecificity can significantly influence toxicity, stereoisomer-specific data is often not tested, predicted by models or captured in databases. Accordingly, the 180 cooling agents with unique 2-D structures were used to identify toxicity endpoints in HCM. In the future, a comprehensive toxicological risk assessment of cooling agents would benefit from the integrating multiple computational models, with stereoisomer-specific and experimental studies to understand dose-response relationships, exposure routes, and mechanisms of action.

In summary, we profiled chemicals currently available as potential cooling agents that could be utilized by manufacturers to maintain the effect and appeal of a new generation of menthol-substituted tobacco products. Through continued innovation, many more coolants continue to be designed as potential tobacco product additives. The recent Surgeon General report discusses the chemosensory, physiological, and genetic factors related to cooling agents that can contribute to tobacco product use disparities [23]. Our work complements and expands beyond the findings of the Surgeon General’s report. Using computational methods, we have captured information surrounding the structural, sensory, and toxicity profiles for the potential menthol substitutes and the distinctions that can be made from menthol. Information across sources may not always be consistent in terminology, reporting detail, or experimental rigor. Accordingly, this manuscript should be interpreted as a structured overview of currently available information rather than a definitive evaluation of cooling agents. Although our cooling agent library is by no means an exhaustive collection of all cooling agents available for use in tobacco products, it represents the first comprehensive surveillance-oriented inventory integrating structural characteristics, physicochemical properties, sensory data, TRPM8 activity, and toxicity information across multiple data sources. Given ongoing chemical innovation and evolving product design, we believe this study provides a relevant resource to inform future tobacco policies. Future updates of the inventory may include revisiting and extracting cooling agents from new patents and literature.

## Methods

### A summary of the overall approach is provided in Fig 5. Selection criteria for a cooling agent

**Fig 5:**
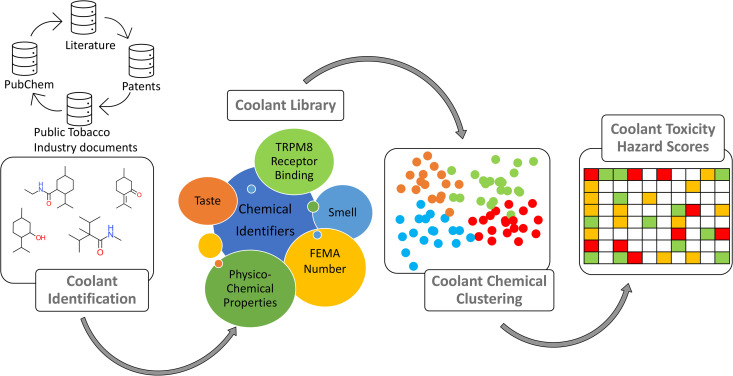
Schema representing methods and data compiled on coolant chemicals including those used in tobacco products. Depicted are data sources and variables, chemical similarity and cluster analysis, and hazard characterization.

We searched multiple public data sources between August 2022 and February 2024 to identify cooling agents. First, we selected a chemical based on the chemical’s flavor profile description as “cool,” “camphor,” “mint,” “peppermint,” and/or “spearmint” in the Flavor and Extract Manufacturers Association (FEMA) ingredient library (https://www.femaflavor.org/), cross-referenced in PubChem Classification (https://pubchem.ncbi.nlm.nih.gov/; PubChem Compound TOC: FEMA Flavor Profile). This search identified 101 chemicals specifically encompassing cool/cooling/mint/camphor FEMA profiles. Next, we expanded the search to include published literature, patents, and Truth Tobacco Industry Documents (more details provided below, and as **Supplemental Data File**) using a snowball search technique starting with context terms such as “menthol-like”, “menthol-substitutes”, “cooling agents”, and “Wilkinson Sword” (WS) to identify additional cooling agents and gather information on their reported cooling attributes. This search identified an additional 127 chemicals, including 20 with a FEMA number. In vitro cell-based assays that overexpress TRPM8 receptors are often used to identify cooling agents. In an iterative process, sensory perception patent searches in Google patents (https://patents.google.com/) and TRPM8-related patents (https://pubchem.ncbi.nlm.nih.gov/#query=trpm8&tab=patent&page=2&selected_id_type=cid) identified patents that describe new chemicals with cooling properties, specifically those used in tobacco products (Supplemental Data File). Chemicals without independent intrinsic cooling activity and whose sole documented function is thermal decomposition to menthol were excluded from the library. We searched Truth Tobacco Industry Documents (https://www.industrydocuments.ucsf.edu/tobacco/) using the above-mentioned search terms to identify cooling agents and gain more insight about industry consideration of their use in commercial tobacco products. Although patents and Truth Tobacco Industry Documents are not peer-reviewed, conclusive, or consistent across inventors and manufacturers, they provide valuable insights into themes surrounding cooling agents. The list of chemicals and references are provided in the **Supplemental S1 Data File**.

Some of the chemicals identified in the peer-reviewed literature, patent applications, and Truth Tobacco Industry Documents only had structural drawings and unconventional chemical names without other common chemical identifiers. In many such cases, stereoisomer-specific data are missing. In cases where several derivatives existed, we tentatively captured only representative parent structures or backbone structures from patents. We used ChemDraw version18.0 (PerkinElmer, Inc., CT) software and FDA-Global Substance Registration System (GSRS; https://gsrs.ncats.nih.gov/) [59] to convert some chemical names to structures and to extract available information in PubChem. Removal of salts and stereoisomers was performed using Canvas version 2020, 3.9 software (Schrödinger Inc., NY) with cheminformatics simulations based on quantum and classical physics [60]. After initial extraction, a chemist manually reviewed and curated data related to chemical identities and structures.

For each cooling agent, we extracted available chemical identifiers from PubChem, including chemical name, PubChem Compound ID (PubCID), Chemical Abstracts Service registry numbers (CASRNs), chemical structural information in the form of isomeric Simplified Molecular-Input Line-Entry System (SMILES) code, IUPAC International Chemical Identifier Key (InChIKey) and FEMA profile for “generally recognized as safe” (GRAS) flavorings “under conditions of intended use in foods.” We collated information on the cooling profiles of the agents, as reflected by either TRPM8 receptor binding and/or sensory cooling perception studies, from published literature, patents, and Truth Tobacco Industry Documents. One of the main challenges in aggregating this data was the lack of unique chemical identifiers represented across all data sources, and thus information had to be collated to include multiple chemical identifiers, including one that represents the chemical structure (SMILES). We excluded chemicals from the library if they were inorganic; mixtures; essential oils such as peppermint and spearmint; or had errors in the SMILES syntax.

The unique 2-D chemical structures were used as the ultimate identifier for further computational analysis of physico-chemical properties and toxicity since many chemicals in the library lack stereo-specificity and the predictive models utilize only 2-D molecular structures for the calculations. The physico-chemical property descriptors based on SMILES codes for the 180 unique 2-D chemical structures were predicted using default settings in Canvas software. The software utilizes 2-D molecular structures for calculations of physico-chemical properties and does not take stereospecificity into consideration. We conducted similar analyses using publicly available prediction tools (OECD QSAR Toolbox and EPA’s EPI Suite™, data not shown) which demonstrated overall concordance between the methods for the predicted physico-chemical properties.

### Cooling agent chemical space and chemical clustering

To classify the structural properties of cooling agents in the library based on manual clustering of the data by the main structural features, we were able to broadly group chemicals based on four main chemical skeletons (*p*-menthane, bicyclic bridged/ fused, substituted amides and all other miscellaneous groups; S1 Table).

To further organize the cooling agent library, we grouped the unique 2-D chemical structures based on their structural similarities using unsupervised machine learning and hierarchical clustering techniques, as described by Abdel-Shafy et al. [42] The chemical structure information represented by SMILES was first used to calculate extended connectivity type molecular fingerprints (hashed circular fingerprints with a default length of 1024, taking rings and atomic properties into account allowing for specificity and efficiency in capturing infinite molecular features) [61]. The extended connectivity fingerprints are well-suited for similarity searching and clustering, and structure activity relationship modeling. The structural attribute information represented by extended molecular fingerprints was then used to generate a Tanimoto distance matrix to calculate the chemicals’ structural dissimilarity. The dissimilarity matrix was converted to a multidimensional space and processed using the Knowledge Discovery by Accuracy Maximization (KODAMA) unsupervised algorithm [43,62] with 100 iterative processes and 50 iterative cycles and with 20 repetitions of KODAMA analysis, thereby maximizing cross-validated accuracy. The output generated by KODAMA is a two-dimensional plot representing the “chemical distances” between all the cooling agents. The optimal number of clusters was estimated to be six from the Rousseeuw’s Silhouette quality index (S1 Fig), which graphically aids the interpretation and validation of cluster analysis and in selecting the optimal number of clusters [63]. Cooling agent hierarchical clustering was then built on the KODAMA output using the complete linkage agglomeration method, which identifies similar chemicals. The unsupervised KODAMA algorithm, Rousseeuw’s Silhouette quality index, and hierarchical clustering were all implemented via openly available *R* package *MetChem* (version 0.4) [42,64] in R Studio (Version 2022.12.0.353), graphs generated using ggplot2 [65] and ggbreak [66].

### Toxicity and hazard assessment of cooling agents using ePA’s hazard comparison module

To rapidly assess potential human health effects and ecotoxicity, we generated hazard profiles for the cooling agents using EPA’s web-based Hazard Comparison Module (HCM, https://www.epa.gov/comptox-tools/cheminformatics; version: DEV, build: 2023-03-09). This publicly available cheminformatics tool compiles hazard records of experimental and quantitative structure-activity relationship (QSAR) predictions from multiple data sources and converts them into scores (inconclusive, low, medium, high, or very high) for various human health effects and ecotoxicity endpoints. The integrated score for each endpoint in the HCM is based on the highest hazard record from the most authoritative data source [67]. HCM prediction models may not capture stereoisomer-specific data. Thus, to avoid data discrepancies, we input the cooling agent’s CASRN or, if CASRN was not available, SMILES code into HCM to generate a hazard profile for each of the 180 unique 2-D structure chemicals. The 15 human health effects endpoints available in HCM at the time of our assessment were acute mammalian toxicity (oral, inhalation, and dermal), carcinogenicity, genotoxicity and mutagenicity, endocrine disruption, reproductive toxicity, developmental toxicity, neurotoxicity (repeat and single exposure), systemic toxicity (repeat and single exposure), skin sensitization, skin irritation, and eye irritation. In addition, HCM provides 2 ecotoxicity (acute and chronic aquatic toxicity) endpoints.

## Supporting information

S1 Data File 228 cooling agent chemical library with chemical identifiers, computationally predicted physico-chemical properties, sensory cooling perception data, TRPM8 binding data, tobacco product ingredients, hazard profiles with associated hazard records for the 180 unique 2-D structure cooling agents, reference to Truth Tobacco Industry Documents, literature and patents used to identify chemicals with cooling properties. (XLSX)

S1 Table The structural skeletons associated with the cooling agents based on manual clustering of the data by the main structural feature/functional group. (DOCX)

S1 Fig The optimal number of chemical clusters estimated from the Rousseeuw’s Silhouette quality index based on the dissimilarity matrix of 180 unique 2-D chemical structures. (DOCX)

## Disclaimer

The findings and conclusions in this report are those of the authors and do not necessarily represent the official position of the Food and Drug Administration. Schrodinger software package was made available to FDA-CTP through licensing agreements with the company (Contract number 75F40120C00059). The mention of commercial products, their sources, or their use in connection with material reported herein is not to be construed as either an actual or implied endorsement of such products by FDA. All other software used are publicly available.
