# Supplementary material for: The tip of the iceberg: Profiling cooling agents using computational approaches to inform tobacco regulatory science
Source: PLoS One. 2026 Apr 16;21(4):e0346126. doi: 10.1371/journal.pone.0346126 (PMC13086334; doi:10.1371/journal.pone.0346126)
Supplement: S1 Table — (DOCX) [file pone.0346126.s002.docx]

**Table S1**: The structural skeletons associated with the cooling agents based on manual clustering of the data by the main structural feature / functional group.

| **Name** | **Skeleton** | **#** | **Structural features** | **General structure** |
| --- | --- | --- | --- | --- |
| *p*-Menthane | 1 | 133 | substituted p-menthane, including Menthol |  |
| Bicyclic and bridged / Bicyclic and fused | 2 | 32 | substituted bicyclic and bridged/ fused ring type, such as substituted bornane, pinene, thujane, and dehydro-azulene |  |
| substituted methyl-diisopropylacetamide | 3 | 13 | substituted methyl-diisopropylacetamide |  |
| Miscellaneous | 4 | 50 | Miscellaneous group including aromatic, aliphatic, cyclic, and heterocyclics; with one or multiple functional groups such as alcohols, ethers, aldehydes, ketones, esters, amines, amides, hydrazides, nitro, nitrile, acetals/ketals | Not Applicable |
