# Supplementary material for: The tip of the iceberg: Profiling cooling agents using computational approaches to inform tobacco regulatory science
Source: PLoS One. 2026 Apr 16;21(4):e0346126. doi: 10.1371/journal.pone.0346126 (PMC13086334; doi:10.1371/journal.pone.0346126)
Supplement: S1 Fig — (DOCX) [file pone.0346126.s003.docx]

**S1 Fig**: The optimal number of chemical clusters estimated from the Rousseeuw’s Silhouette quality index based on the dissimilarity matrix of the 180 unique 2-D chemical structures.
